# Improved mitochondrial amino acid substitution models for metazoan evolutionary studies

**DOI:** 10.1186/s12862-017-0987-y

**Published:** 2017-06-12

**Authors:** Vinh Sy Le, Cuong Cao Dang, Quang Si Le

**Affiliations:** 1 University of Engineering and Technology, Vietnam National University Hanoi, Hanoi, Vietnam; 2 School of Pharmacy and Biomedical Sciences, University of Portsmouth, Winston Churchill Avenue Portsmouth, Portsmouth, PO1 2UP UK

**Keywords:** Mitochondrial amino acid substitution models, Metazoa, Vertebrates, Invertebrates

## Abstract

**Background:**

Amino acid substitution models play an essential role in inferring phylogenies from mitochondrial protein data. However, only few empirical models have been estimated from restricted mitochondrial protein data of a hundred species. The existing models are unlikely to represent appropriately the amino acid substitutions from hundred thousands metazoan mitochondrial protein sequences.

**Results:**

We selected 125,935 mitochondrial protein sequences from 34,448 species in the metazoan kingdom to estimate new amino acid substitution models targeting metazoa, vertebrates and invertebrate groups. The new models help to find significantly better likelihood phylogenies in comparison with the existing models. We noted remarkable distances from phylogenies with the existing models to the maximum likelihood phylogenies that indicate a considerable number of incorrect bipartitions in phylogenies with the existing models. Finally, we used the new models and mitochondrial protein data to certify that Testudines, Aves, and Crocodylia form one separated clade within amniotes.

**Conclusions:**

We introduced new mitochondrial amino acid substitution models for metazoan mitochondrial proteins. The new models outperform the existing models in inferring phylogenies from metazoan mitochondrial protein data. We strongly recommend researchers to use the new models in analysing metazoan mitochondrial protein data.

## Background

An amino acid substitution model (model for short) includes a 20 × 20 matrix and an amino acid frequency vector. The matrix represents the instantaneous substitution rates among amino acids while the amino acid frequency vector serves as the equilibrium frequencies of the 20 amino acids. The substitution rates characterise the biological, chemical, and physical correlations among amino acids [1]. Amino acid substitution models are the key to infer phylogenies from protein data. Distance-based methods use amino acid substitution models to estimate pairwise distances among sequences, while maximum likelihood or Bayesian methods require amino acid substitution models to calculate the likelihood of data [2].

Estimating amino acid substitution models is much more challenging than estimating nucleotide substitution models due to a large number of parameters to be optimised. For example, the general time reversible model for nucleotides contains 8 parameters in comparing to 208 parameters for models of amino acid substitutions. Thus, amino acid substitution models are typically estimated from large datasets.

It is well established that models of different species or protein types would be diverse [3–5]. For example, Dang et al. showed that the model for influenza proteins is highly different from general models [3]. Note that protein structures also contribute to amino acid evolution patterns [6, 7].

Mitochondria (mt) are energy factories and play an essential role in supplying cellular energy [8]. The mitochondrial genome encodes 13 proteins that are widely used to infer phylogenies [7, 9–12]. Few groups have estimated empirical models from mt protein data (mt models). Adachi and Hasegawa were the first to estimate an mt model, named mtREV, from 20 complete vertebrate sequences [13]. They argued that the difference between the universal code and the mitochondrial code might be partially responsible to the difference between amino acid substitution patterns from nuclear and mitochondrial-encoded proteins. Abascal et al. built another mt model, mtArt, from 36 arthropod species to analyse the data of invertebrate species [14]. Note that although invertebrates are paraphyletic, the term *invertebrates* is widely used as a convenient shorthand in communication [5, 13–15]. Neither mtREV nor mtArt is appropriate for datasets consisting of diverse metazoan lineages, as they were specifically estimated from either vertebrate or invertebrate protein data. Rota-Stabelli et al. solved the problem by introducing an mt model (mtZoa) estimated from 117 general metazoan species [5]. They recommended to use mtZoa for analysing datasets from diverse or basal metazoan groups. The existing mt models (mtREV, mtArt, and mtZoa) outperform general models (e.g., LG [16] and WAG [17]) in inferring phylogenies from mt protein data, even though they were estimated from small datasets.

The main issue of the existing mt models comes from their small training datasets of at most 117 species. This was due to the limited mt protein data available and the capability of estimation methods at the time these studies were carried out. Consequently, the models might over-fit to training data due to a large number of free parameters of the amino acid substitution model (precisely 208 free parameters). In other words, the existing models may fit too well to training sequences but poorly represent others. Above all, the existing mt models cannot appropriately represent nearly a million available mt protein sequences of more than 34 thousands metazoan species, as they were estimated from only a limited number of species.

In this paper, we introduce new mt models for metazoan and vertebrates. Although invertebrates are not monophyletic, their mitochondria have the same genetic codes. The genetic codes of invertebrate mitochondria are different from that of vertebrate mitochondria. The difference might result in different amino acid substitution patterns from invertebrate and vertebrate mitochondrial-encoded proteins [5, 13, 14]. Therefore, we also introduce a new mt model for invertebrates. To this end, we created three datasets from 125,935 mt sequences of 13 proteins from 34,448 metazoan species. Then, we implemented the fast and accurate method, FastMG [18], to estimate three new mt models from these three datasets.

We validated the new models by assessing the likelihood of phylogenies with the new models for both training and testing data. We summarised the experimental results to show the advantage of the new models in inferring the maximum likelihood phylogenies (called the best phylogenies) in comparison to existing mt models. Experimental results revealed remarkable distances from the phylogenies with the existing models to the best phylogenies. We proved that the remarkable distances imply a considerable number of incorrect bipartitions in the phylogenies with the existing models. Although we could not evaluate the topological quality of phylogenies with the new models, as they were often the best phylogenies, we would expect significant topological improvement due to their large likelihood advantage over the phylogenies with the existing models.

Finally, we applied the new models to tackle a debated question about the location of Testudines within amniotes. We used IQ-TREE with the new models to build the maximum likelihood phylogeny of 993 amniotes from their mt protein data. We learned from the phylogeny that Testudines, Aves, and Crocodylia form one separated clade within amniotes.

## Results and discussion

### Data preparation

We downloaded all mt protein sequences of 34,448 species in the metazoan kingdom from NCBI (National Center for Biotechnology Information, 2016) and then mapped them onto 13 mt proteins. We selected one sequence per species to eliminate bias on intensively studied species (e.g., 30,000 human sequences). As the result, we obtained 125,935 sequences to form three datasets for metazoan, vertebrate, and invertebrate categories. We kept all sites, as removing sites with missing data would lead to worse phylogenies [19]. We divided each dataset into a training dataset and a testing dataset containing 90% and 10% of sequences, respectively.

We implemented the fast and accurate method, FastMG [18], to estimate three new mt models, *mtMet, mtVer*, and *mtInv* from metazoan, vertebrate, and invertebrate training datasets, respectively. As FastMG is infeasible for alignments of several thousands sequences, we split alignments based on the taxonomy tree to obtain sub-alignments of at most one thousand sequences. Then we divided these sub-alignments into smaller sub-alignments of at most 128 sequences using the tree-based splitting algorithm in FastMG. In addition, we removed branches with lengths equal to zero or larger than two in order to eliminate data noise. The data are summarised in Tables 1 and 2. Note that the FastMG algorithm starts from an initial model and iteratively optimises the model until the likelihood improvement is insignificant.

**Table 1:** The number of sequences of 13 mt proteins for metazoan, vertebrate, and invertebrate datasets

	Metazoan		Vertebrate		Invertebrate	
Protein	Training	Testing	Training	Testing	Training	Testing
ATP6	8493	938	5752	636	2741	302
ATP8	8412	928	5726	632	2686	296
COX1	7090	784	4633	512	2457	272
COX2	9363	1033	5023	555	4340	478
COX3	6867	759	4208	466	2659	293
CYTB	12,894	1422	10,326	1139	2569	282
ND1	8280	912	5355	590	2926	321
ND2	14,541	1597	11,885	1306	2655	292
ND3	9074	997	6262	687	2812	310
ND4	7191	793	4567	503	2625	289
ND4L	7274	803	4498	496	2776	307
ND5	6975	769	4409	487	2566	282
ND6	6977	769	4360	480	2617	289
Total	125,935		85,493		40,442	

Each dataset is divided into a training dataset and a testing dataset with a 9 to 1 ratio

**Table 2:** The number of sequences, alignments, and sites in metazoan, vertebrate, and invertebrate training and testing datasets

	Training			Testing		
	#Sequences	#Alignments	#Sites	#Sequences	#Alignments	#Sites
Metazoan	103,637	1155	362,062	12,701	139	47,477
Vertebrate	68,536	772	238,429	8878	95	29,999
Invertebrate	35,089	390	125,849	3908	48	17,792

### The fit of new models to training datasets

We measured the fit of new models to the training datasets. Table 3 shows significant likelihood improvements of the new models over the initial model, mtZoa, for metazoan, vertebrate, and invertebrate training datasets. The first iteration contributed about 99% of the total likelihood improvement. The optimisation process was terminated after the third iteration, as the gain from the third iteration was insignificant.

**Table 3:** Total log-likelihood of the target function (Eq. 1) on training datasets

	Metazoan	Vertebrate	Invertebrate
mtZoa (initial model)	−1.23427e + 07	−5.50036e + 06	−6.85299e + 06
First iteration	−1.21987e + 07	−5.32959e + 06	−6.77590e + 06
Second iteration	−1.21987e + 07	−5.32671e + 06	−6.77536e + 06
Third iteration (final model)	−1.21987e + 07	−5.32671e + 06	−6.77536e + 06
AIC/site	0.795	1.456	1.232
BIC/site	0.790	1.430	1.220

AIC/site (BIC/site) is the AIC (BIC) improvement per site of the final model in comparison to the initial model mtZoa

There is no likelihood improvement after two iterations

The better Akaike and Bayesian information criterion scores [20, 21] of the new models in comparison to the initial model, mtZoa, confirm the better fit of the new models to the training data. The scores guarantee that the likelihood gain of the new models comes from their genuine fit and overwhelm the penalty of free parameters.

### Model analysis

**Fig. 1:**
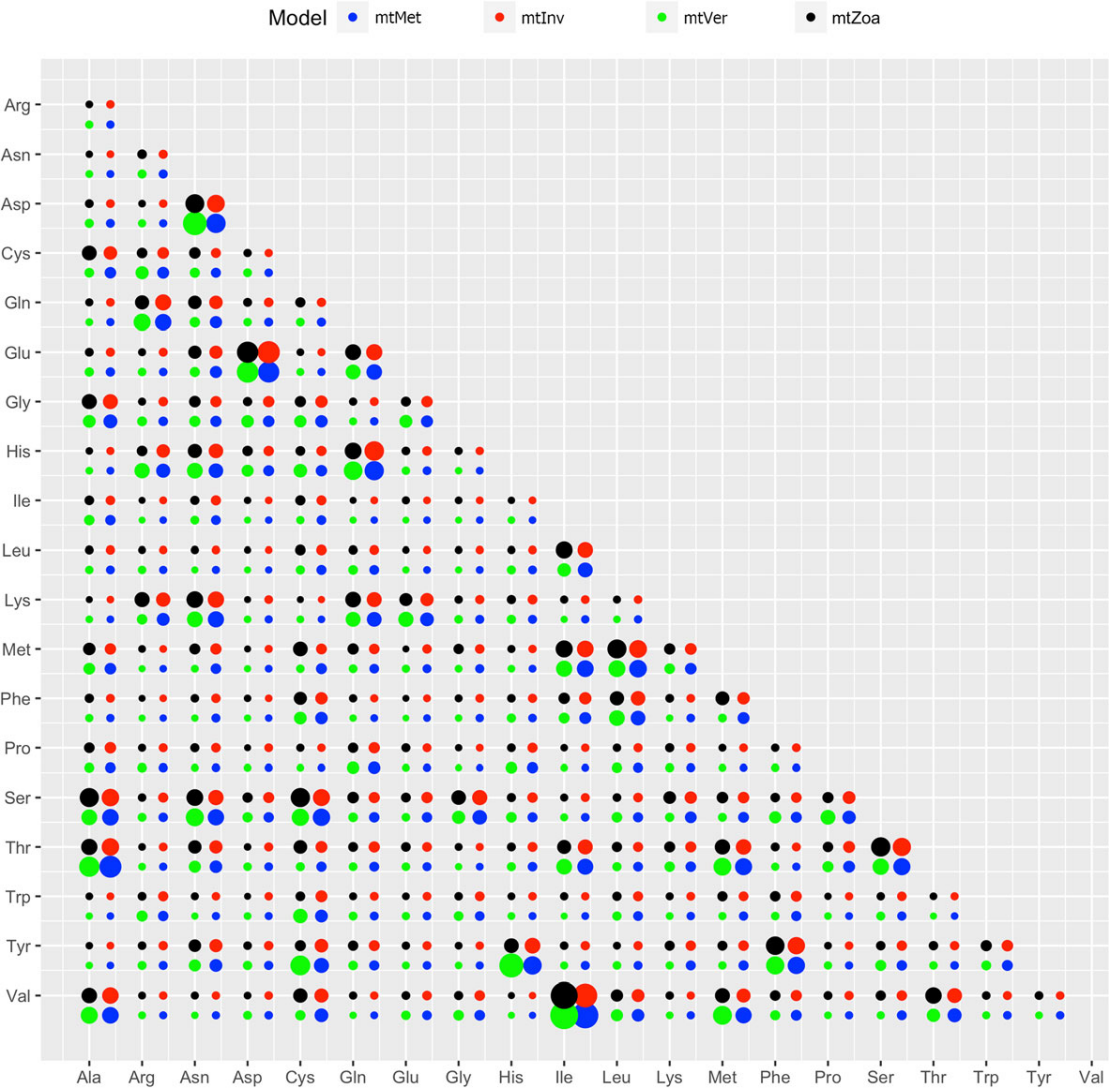
Amino acid exchangeability rates of the mtMet, mtInv, mtVer, and mtZoa models. There are some considerable difference between mtZoa and the new models

**Fig. 2:**
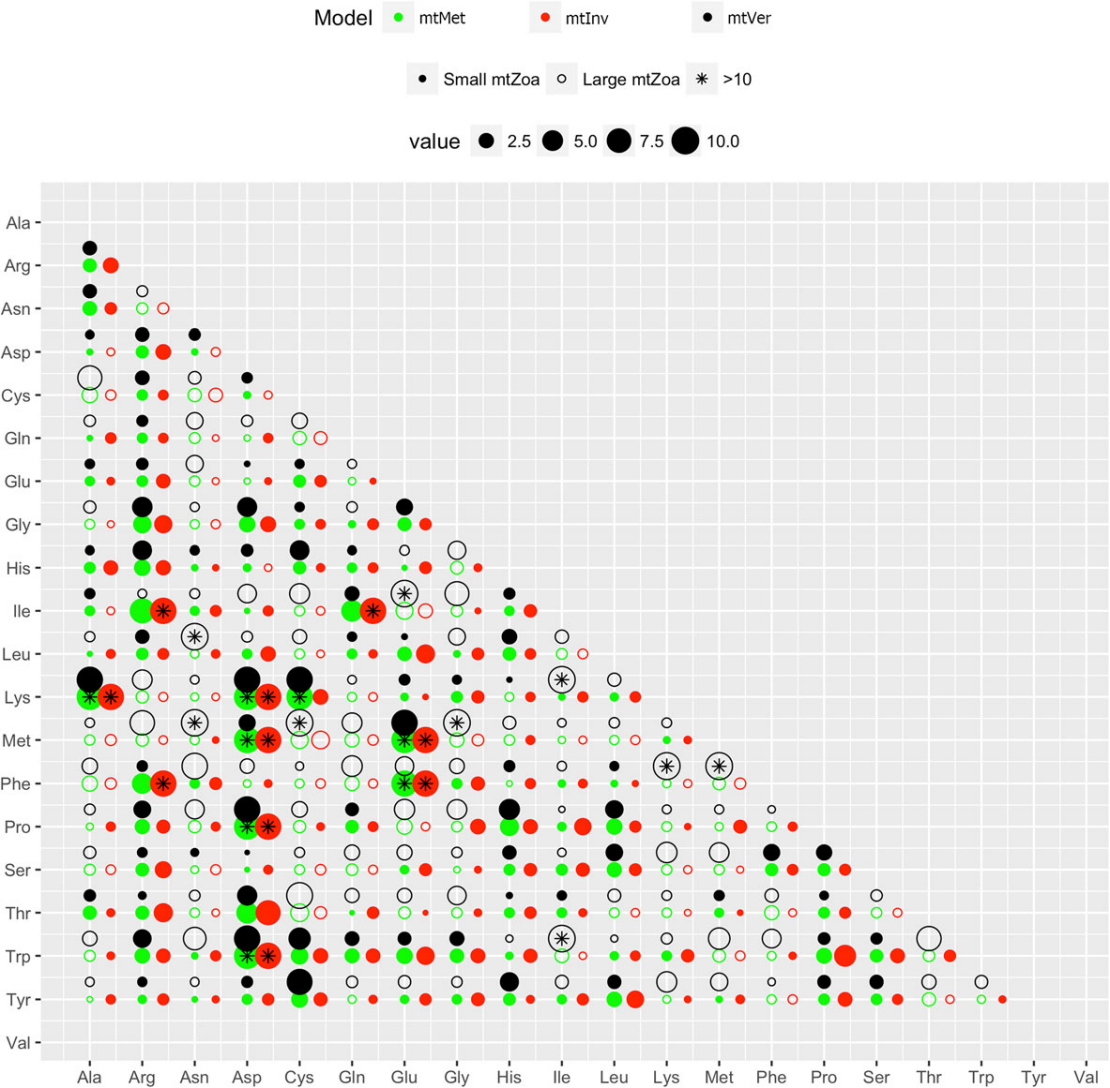
The ratio of exchangeability rates between mtZoa and mtMet/mtVer/mtInv models. The size of one circle represents the exchangeability rate between mtZoa and other models. The solid (unfilled) circles represent exchangeability rates where mtZoa is smaller (bigger) than the three models. For visualization, the large ratios are trimmed at 10 and marked with ‘*’

**Fig. 3:**
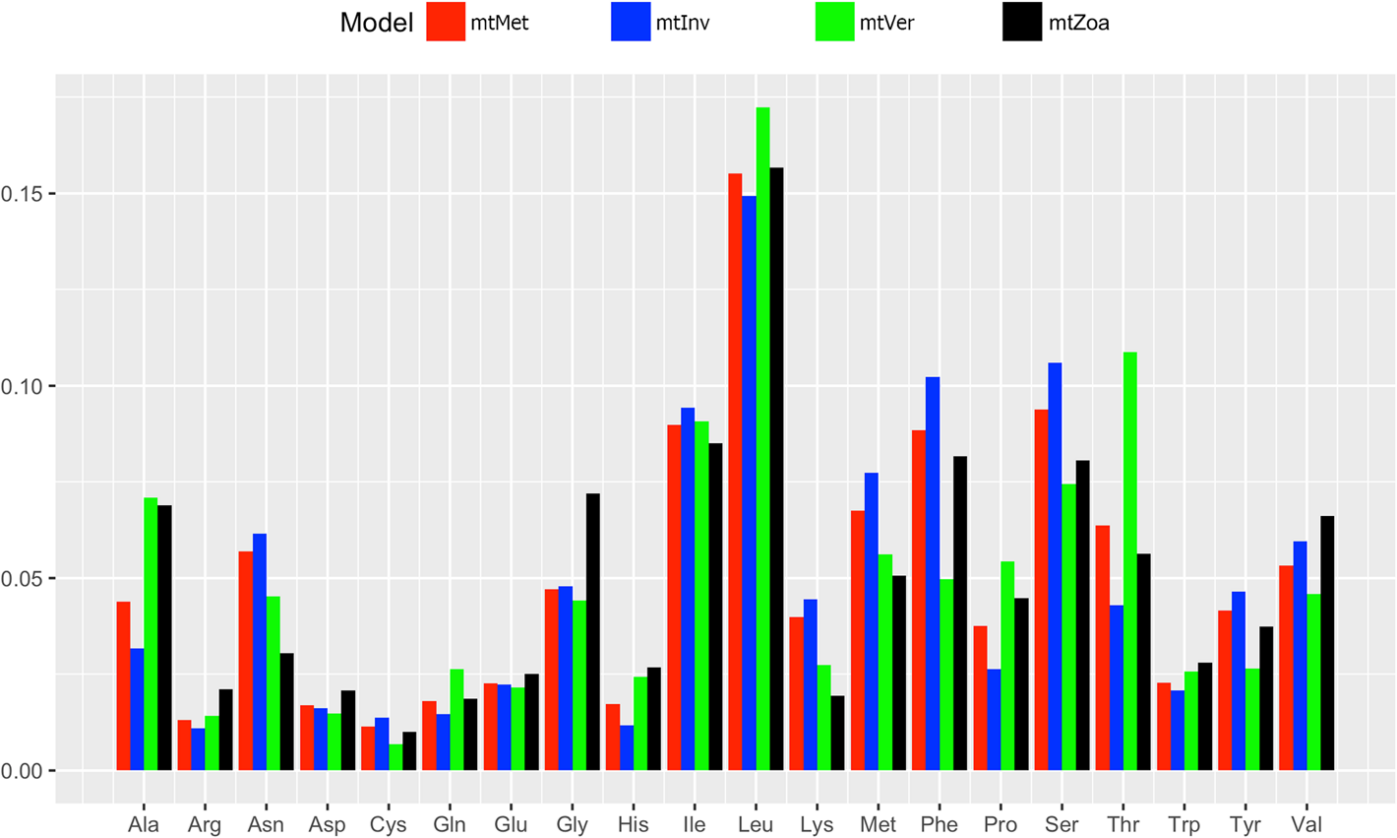
Amino acid frequencies of the mtMet, mtInv, mtVer, and mtZoa models. There are some considerable difference between mtZoa and the new models

Figures 1 and 2 show significant differences in exchangeability patterns between amino acids among the four models: mtZoa, mtMet, mtVer, and mtInv (see). For example, the exchangeability rate between *methionine* and *glutamine* in mtMet is about 10 times greater than that in mtZoa (0.155 vs 0.0016). The exchangeability rate between these two amino acids in mtVer is a third of that in mtInv (0.075 vs 0.228). Figure 3 shows a clear variety of amino acid frequencies among the four models, especially between mtVer and mtInv). For instance, the frequency of *Threonine* in mtVer is about three times as much as that in mtInv (0.146 vs 0.0428).

The low pairwise correlations of exchangeability rate matrices (or frequency vectors) of the mt models confirm high varieties among the models (Table 4). The mtInv and mtVer models are the most diverse pair with the smallest correlation of exchangeability rates (0.775). Note that the correlation between the two popular general models LG and WAG is 0.912. As expected, mtMet is the closest model to mtZoa in terms of exchangeability rates, with a 0.929 correlation score, as both were trained from the metazoan data. Interestingly, mtMet is closer to mtInv than mtVer, although the metazoan training dataset consists of less invertebrate data than vertebrate data. The results indicate diverse evolutionary processes among lineages in the metazoan kingdom.

**Table 4:** Correlations between four models: mtMet, mtInv, mtVer, and mtZoa

	mtMet	mtInv	mtVer	mtZoa	LG	WAG
mtMet		0.976	0.89	0.929	0.527	0.439
mtInv	0.959		0.775	0.875	0.457	0.363
mtVer	0.94	0.866		0.893	0.591	0.529
mtZoa	0.92	0.956	0.829		0.619	0.587
LG	0.837	0.887	0.787	0.894		0.912
WAG	0.825	0.878	0.778	0.85	0.961	

The values in the top triangle represent the correlations between exchangeability matrices, while values in the low triangle are the correlations between frequency vectors

We observed remarkably low correlations between mt models and general models (e.g., the 0.46 correlation score between mtInv and LG). The low correlations imply considerably diverse evolutionary patterns between mt proteins and general proteins. Thus, general models are not an appropriate choice in inferring phylogenies from mt protein data.

### Likelihood improvement on testing alignments

We assessed the performance of the new mt models (mtMet, mtVer, and mtInv) and the existing mt models (mtZoa, mtREV, and mtArt) on building maximum likelihood phylogenies. To this end, we used IQ-TREE [22] to build phylogenies with different models on the metazoan, vertebrate, and invertebrate testing datasets. For each testing alignment *D* and a model *M*, we optimised parameters of the rate heterogeneity model (i.e., proportion of invariable sites and shape of Gamma distribution with 4 categories), but fixed the exchangeability rates and base frequencies of the model *M*.

**Fig. 4:**
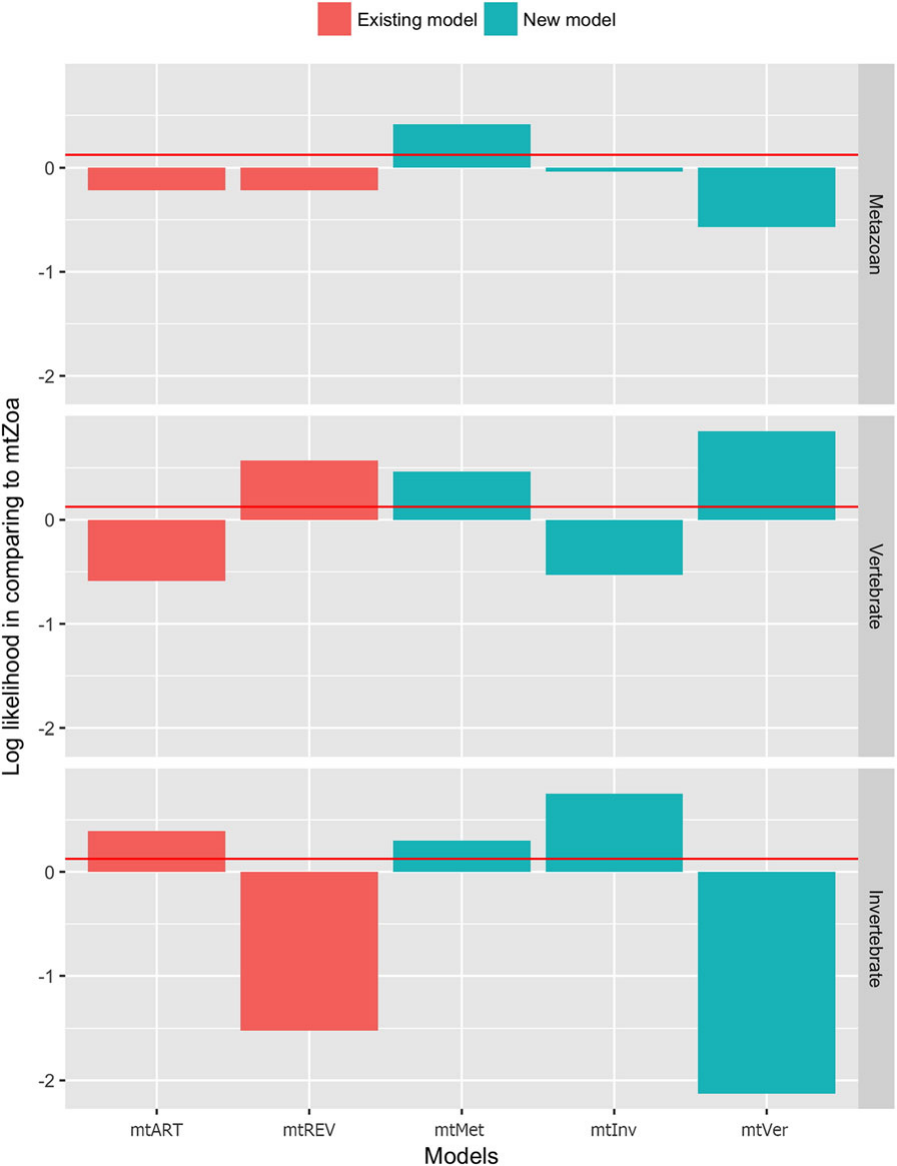
Difference per site between log-likelihood of phylogenies with mtZoa and that with the existing models (mtREV and mtArt), and the new models (mtMet, mtVer, and mtVer). The red line represents the improvement of LG from WAG

It is clear from Fig. 4 that the new models outperform the existing models for all three testing datasets. They are the best-fit models for their corresponding testing data (e.g., mtMet is the best-fit model for the metazoan testing data). Note that the second-best fit model for a certain testing dataset is the existing model estimated from the training data of the same category as the testing dataset (e.g., mtZoa is the second-best fit model for the metazoan testing data). The log-likelihoods of the phylogenies with the new models are significantly higher than those of the existing models. For example, the likelihood advantage of mtMet to the second-best model, mtZoa, on the metazoan testing data is about 0.41 log points per site (or 1640 log points for a concatenated alignment of 4000 sites). This improvement is about four times as much as the improvements of LG from WAG [16]. In short, the three new models outperform the three existing models in their corresponding categories.

**Fig. 5:**
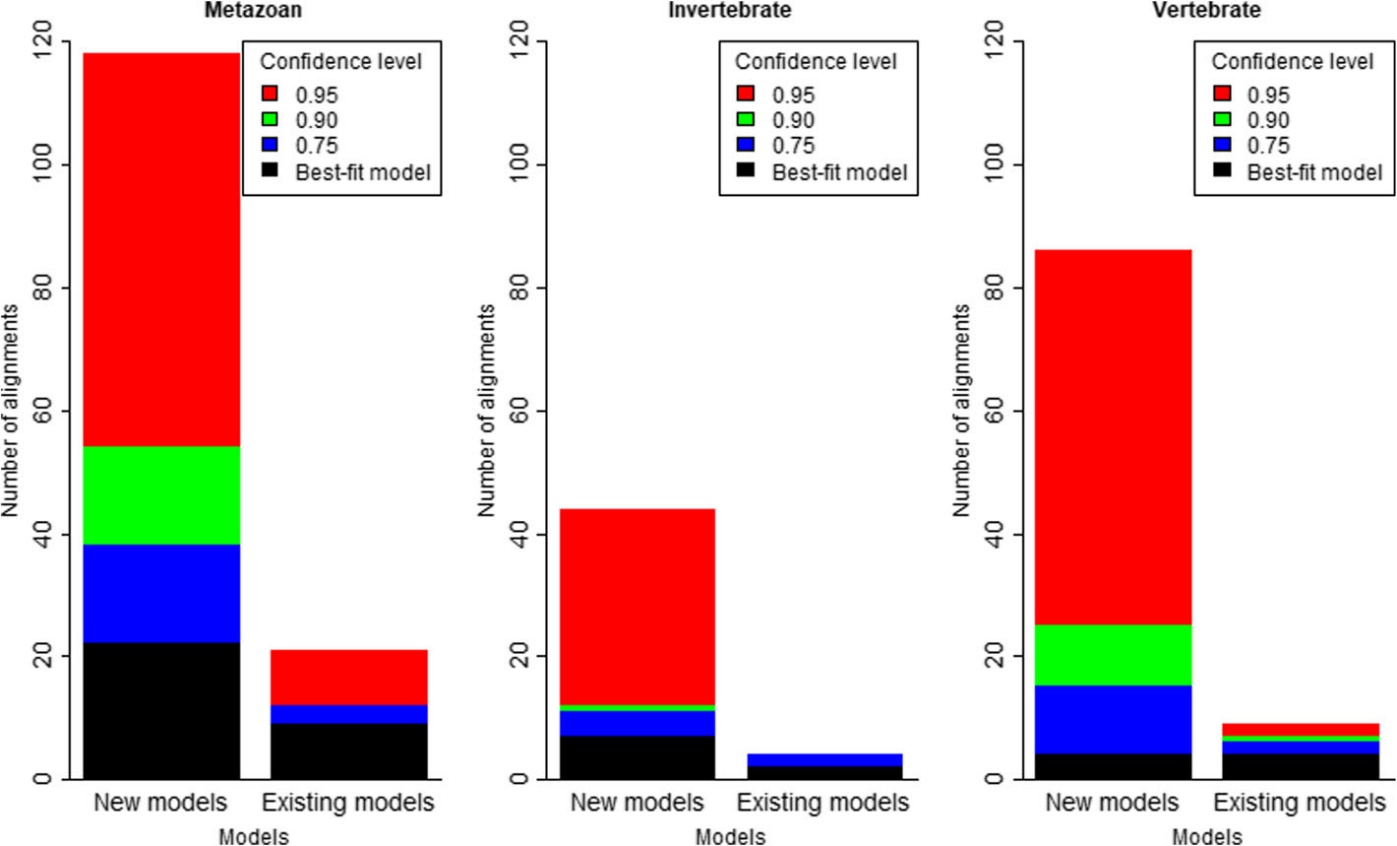
We used the approximately unbiased SH test to compute the confidence levels for phylogenies with the new and existing models on metazoan, vertebrate and invertebrate testing datasets. For each testing alignment *D*, we computed the site-wise log likelihoods for every (*T*_*i*_, *M*_*i*_|*D*) where *M*_*i*_ is one of six mt models and *T*_*i*_ is the phylogeny of *D* under *M*_*i*_. The CONSEL program was used for assessing the confidence levels for each (*T*_*i*_, *M*_*i*_|*D*)

We analysed the performance of the mt models at the individual alignment level. We used the approximately unbiased SH test [23] to compute confidence levels for phylogenies with the models. Given a testing alignment *D*, we estimated the maximum likelihood tree *T*_*i*_ according to model *M*_i_ where *M*_i_ is one of the six mt models. We computed the site-wise log likelihoods for every (*T*_*i*_, *M*_*i*_|*D*), and subsequently used the CONSEL program [24] for assessing their confidence levels. The approximately unbiased SH test helps us to confirm whether the likelihood improvement comes from models and trees or from artefacts of numerical analyses in IQ-TREE. Figure 5 confirms the advantage of the new models in inferring phylogenies for all three testing datasets. The new models demonstrate a better fit for almost all testing alignments in comparison with the existing models (e.g., 85 out of 95 vertebrate alignments). The approximately unbiased SH test also confirms the superiority of the new models with high confidence levels (e.g., 67 out of 95 vertebrate alignments at the 0.9 confidence level). The existing models are still the best-fit models for some alignments, but only significantly better than the new models in a few cases. For example, the existing models are the best-fit models for 10 out of 95 vertebrate alignments, but only significantly better for one alignment at the 0.9 confidence level.

**Fig. 6:**
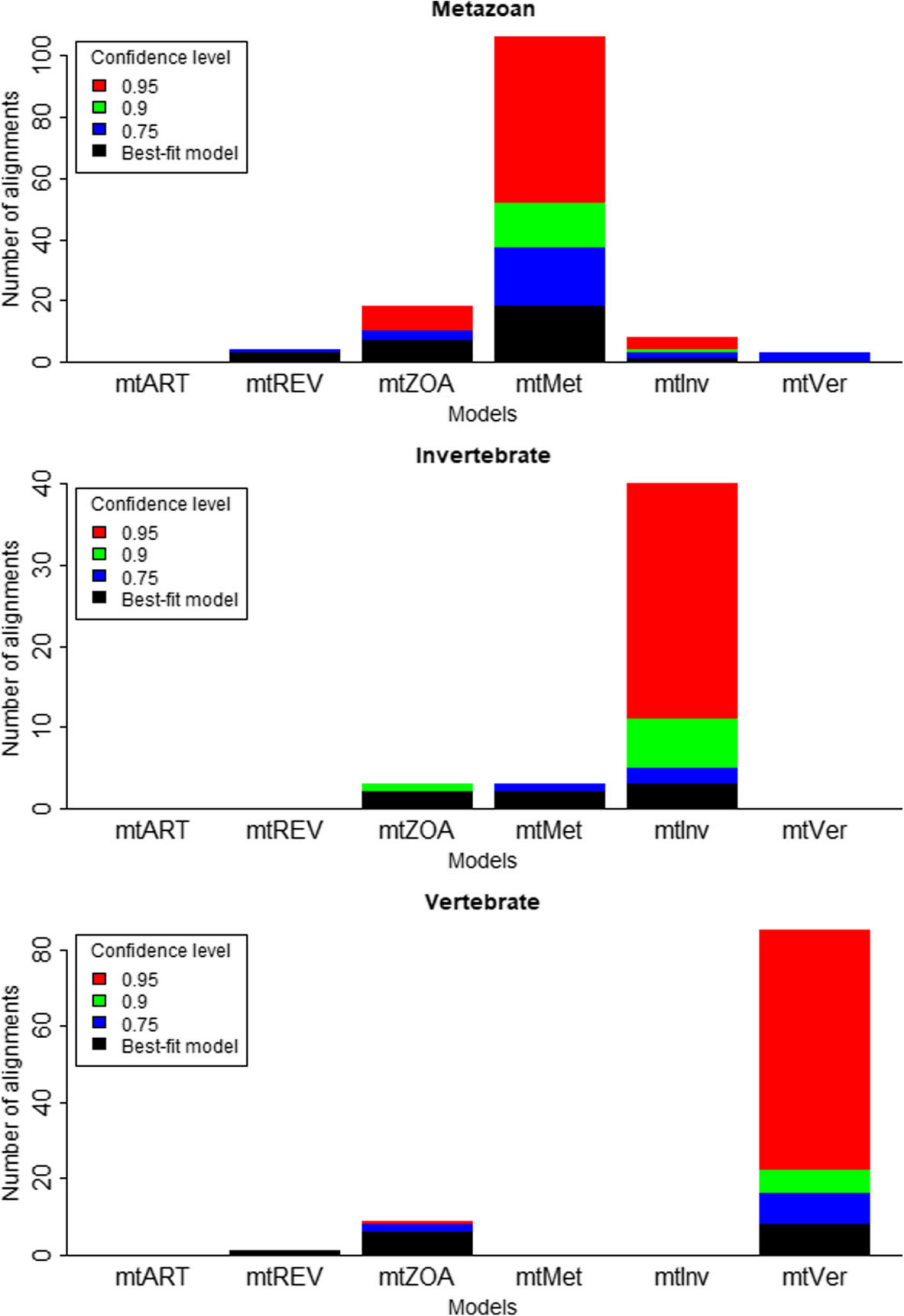
We used the approximately unbiased SH test (explanations are given in Fig. 5) to compute the confidence levels for phylogenies with six mt models (mtMet, mtVer, mtInv, mtArt, mtREV, and mtZoa) on metazoan, vertebrate and invertebtate testing datasets

More specifically, we examined the performance of the six mt models individually (see Fig. 6). We highlight some following findings:

- The best-fit model for a certain testing alignment is typically the one estimated from the training data of the same category as the testing alignment. For example, 85 out of 95 vertebrate testing alignments fit best with mtVer, which was estimated from the vertebrate training data.
- The mtVer model outperforms the mtInv model for all vertebrate testing alignments and vice versa. This is explainable, as the two models are highly diverse. The mtMet model is usually the best-fit model for metazoan testing alignments. However, some metazoan testing alignments are biased on vertebrate or invertebrate species, therefore, mtVer or mtInv might fit better than mtMet for those diverse metazoan alignments.

Finally, we compared the performance of new mt models to LG4X, C60 (site-heterogeneous models) [25] and PHAT (a transmembrane-specific amino acid substitution model) models [26]. Table 5 shows that the new mt models outperformed LG4X, C60 and PHAT models in terms of AIC and BIC.

**Table 5:** The AIC (BIC) per site of nine models on three testing datasets (the smaller AIC (BIC) the better model)

	mtZOA	mtREV	mtArt	LG4X	C60	PHAT	mtMet	mtInv	mtVer
Metazoan	120.049 (122.011)	120.478 (122.440)	120.476 (122.438)	124.613 (126.629)	124.748 (126.710)	132.966 (134.928)	119.216 (121.178)	120.125 (122.087)	120.769 (122.731)
Invertebrate	133.182 (134.831)	136.229 (137.878)	132.394 (134.044)	138.975 (140.675)	137.979 (139.628)	146.924 (148.573)	132.587 (134.236)	131.674 (133.324)	137.432 (139.082)
Vertebrate	97.129 (99.249)	95.979 (98.099)	98.301 (100.421)	99.851 (102.028)	99.040 (101.159)	107.722 (109.842)	96.195 (98.315)	98.180 (100.299)	95.435 (97.555)

Nine models include six mt models, two site-heterogeneous models (i.e., LG4X, C60), and PHAT model (a transmembrane-specific substitution model)

### Phylogeny topology differentiation on testing alignments

We investigated the topological quality of phylogenies with the six mt models by measuring their topological distances from the best phylogenies. Specifically, we used the Robinson­Foulds (RF) metric to measure the distance between two phylogenies, as it represents the number of unique bipartitions in two phylogenies [27]. We learn from Lemma 1 that the lower-bound number of incorrect bipartitions in a phylogeny can be approximated as a quarter of its RF distance from the best phylogeny.

#### Lemma 1.

Given two binary unrooted trees *T* and *T* ^’^ inferred from the same alignment of *n* taxa. The number of incorrect bipartitions in the worse likelihood phylogeny is at least a quarter of the RF distance between *T* and *T* ^’^.

#### Proof:

Let *T*_0_ be the true binary unrooted tree. It is true that *T*, *T* ^’^, and *T*_0_ have the same number of bipartitions, 2*n* − 3 [28].

Let *p* be the number of shared bipartions in both *T* and *T’.* Let *x* and *y* be the number of unique bipartitions in *T* and *T* ^’^, respectively. As *x* = (2*n* − 3) − *p* and *y* = (2*n* − 3) − *p*, *x* must be equal to *y*.

The RF distance between *T* and *T* ^’^ is *x* + *y* or 2*x*.

Let *S* be the set of all bipartitions in *T* and *T’*, and *S* consists of (2*n* − 3) + *x* bipartitions. Since the true tree *T*_0_ has (2*n* − 3) bipartitions, *S* must consist of at least *x* (half of the RF distance) incorrect bipartitions.

**Fig. 7:**
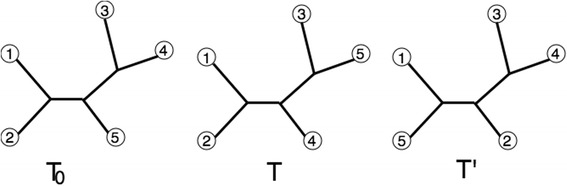
Unrooted binary trees *T* ,  *T* ^′^, and true tree *T*_0_ each has 7 bipartitions. The bipartitions that in *T* but not in *T* ^’^ *is* {(12| 345), (124| 35)}. The bipartitions that in *T* ^’^ but not in *T* is {(15| 234), (152| 34)}. The Robinson and Foulds distance between *T* and *T* ^′^ is four. The set *S* of all bipartitions in *T* and *T* ^′^ is $$ \left\{\begin{array}{c}\left(12|345\right),\left(124|35\right),\left(15|234\right),\kern0.5em \left(152|34\right),\\ {}\left(1|2345\right),\left(2|1345\right),\left(3|1245\right),\left(4|1235\right),\left(5|1234\right)\end{array}\right\}. $$ As the set *S* consists of 2 incorrect bipartitions (i.e., (124| 35) and (15| 234)), the worse tree must contain at least one incorrect bipartition (a quarter of the Robinson and Foulds distance between *T* and *T* ^′^)

Let *T* be the worse likelihood phylogeny. Then , *T* should include at least half of the incorrect bipartitions (*x*/2) as *T* is considered the worse phylogeny. In other words, *T* includes at least a quarter of RF distance between *T* and *T* ^’^. Figure 7 illustrates an example with five taxa.

Table 6 discloses remarkable topological distances from the phylogenies with the three existing models to the best phylogenies. The distances imply a considerable number of incorrect bipartitions in the phylogenies. For example, the phylogenies with mtZoa for metazoan testing alignments contain at least 6.37% incorrect bipartitions (i.e., a quarter of their normalised RF distance from the best phylogenies, 0.255). The results reconfirm the essential role of model selections in inferring phylogenies as a poor model selection (i.e., model and testing data coming from different categories) would lead to low quality phylogenies. The lower-bound numbers of incorrect bipartitions of phylogenies with the new models are indeterminable as they are often the best phylogenies. However, the significant likelihood improvement would expectedly lead to better phylogenies with fewer incorrect bipartitions.

**Table 6:** Normalised Robinson­Foulds (RF) distances between phylogenies with six mt models

		mtArt	mtREV	mtZoa	mtMet	mtInv	mtVer
Metazoan	mtREV	0.323					
	mtZoa	0.243	0.286				
	mtMet	0.307	0.281	0.28			
	mtInv	0.299	0.318	0.293	0.239		
	mtVer	0.353	0.277	0.313	0.276	0.332	
	Best	0.304	0.269	0.255	0.058	0.242	0.277
Vertebrate	mtREV	0.115					
	mtZoa	0.087	0.103				
	mtMet	0.109	0.099	0.100			
	mtInv	0.098	0.104	0.095	0.093		
	mtVer	0.124	0.098	0.114	0.1	0.115	
	Best	0.122	0.096	0.104	0.099	0.112	0.012
Invertebrate	mtREV	0.087					
	mtZoa	0.067	0.082				
	mtMet	0.082	0.075	0.076			
	mtInv	0.08	0.08	0.079	0.064		
	mtVer	0.094	0.076	0.089	0.076	0.087	
	Best	0.081	0.081	0.075	0.064	0.006	0.088

The distances are normalised by dividing by (2n − 3), where *n* is the number of taxa

**Fig. 8:**
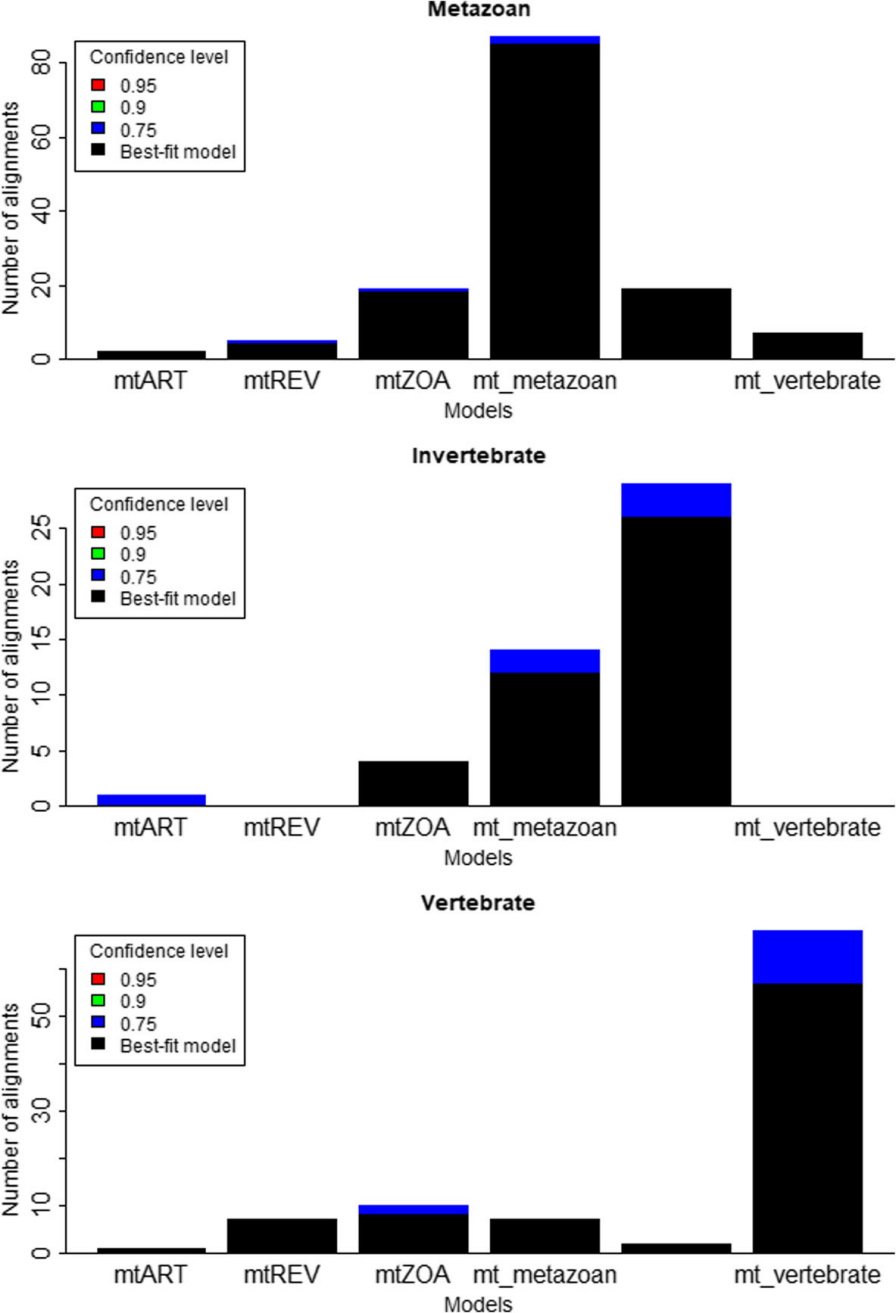
We used the approximately unbiased SH test to examine tree topologies on metazoan, vertebrate and invertebrate testing datasets. For each testing alignment *D*, we determined its best-fit model *M*_*b*_. We fixed tree topologies, but reoptimised other parameters (i.e., branch lengths, parameters of rate heterogeneity model) under the best-fit model *M*_*b*_. Then we used the CONSEL program to assess the confidence levels for every tree topologies

We also applied the approximately unbiased SH test to examine the tree topologies under the best-fit models. Given a testing alignment *D* and its best-fit model *M*_*b*_, we fixed tree topologies, but reoptimised other parameters (i.e., branch lengths, parameters of rate heterogeneity model) under the best-fit model *M*_*b*_. Then we used the CONSEL program for assessing their confidence levels. The test shows that the tree topologies built with the new models are better than that with the existing models in term of likelihood but with lower confidence (Fig. 8). The significant drop of confidence levels reveals that a large proportion of likelihood gain is due to the new models other than tree topologies.

### Location of Testudines within amniotes

**Fig. 9:**
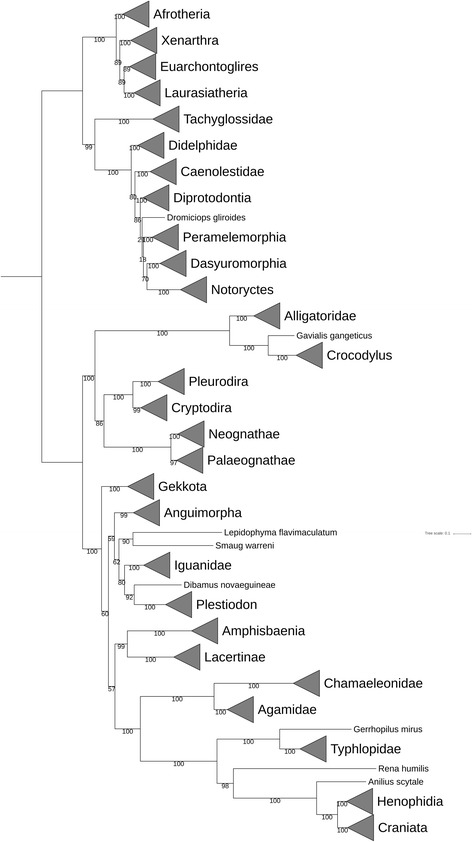
Location of Turtles in Amiphiona. The Testudines clade including two clades (Pleurodira and Cryptodira) is located within the clade of Crocodylia and Aves

We applied the new models to tackle a question about the phylogenetic position of Testudines within amniotes. The question has a long history of debate with at least four hypothesises [29]. To this end, we built a concatenated alignment of 13 proteins for 993 amniotes and used IQ-TREE with all mt, LG4X, and C10 models to infer the best phylogeny, named *T*_*a*_ **(**Fig. 9 **)**. As expected, mtVer resulted in a huge likelihood advantage over other models (i.e., 18,351 log-likelihood advantage over the second-best model, mtMet). We also used a bootstrap method [30] to estimate the reliability of clades in *T*_*a*_.

In general, *T*_*a*_ strongly supports the main clades of the NCBI taxonomy at the family, subfamily, and genus levels. However, the low bootstrap values of some clades at more high levels show the limitation of mt protein data in resolving ambiguous relationships among high level clades.

Specifically, *T*_*a*_ shows strong support (100% bootstrap values) for the clades of the Testudines order, Crocodylia order, and Aves class. In other words, mt proteins contain sufficient phylogenetic signals to correctly place a Testudines, Crocodylia, or Aves species into its corresponding order or class. Moreover, *T*_*a*_ also displays a strong support (100% bootstrap value) for the clade of all Testudines, Crocodylia, and Aves. This means that Testudines, Crocodylia, and Aves form one separated group within amniotes. We validated the finding by moving Testudines out of the clade of Crocodylia and Aves to other positions around. We found that *T*_*a*_ was much better than other phylogenies examined (i.e., better than the second-best phylogeny with 76 log-likelihood points). In other words, Testudines is unlikely to be located elsewhere, rather than within the clade of Crocodilian and Aves. The finding agrees with the conclusion by Crawford et al. [31].

Although *T*_*a*_ shows a strong support for the position of Testudines within the clade of Crocodylia and Aves, unfortunately it cannot determine the exact relationships among them. The low bootstrap value of the clade including Testudines and Aves suggests the uncertainty of the ((Testudines,Aves),Crocodylia) topology. We examined this hypothesis by comparing the topology to two other possible topologies ((Crocodylia,Testudines),Aves) and ((Crocodylia, Aves), Testudines). The tiny likelihood difference among the three topologies implies that none of these topologies really outweighs the others (Table 7). For example, the 0.467 log-likelihood advantage of ((Testudines,Aves),Crocodylia) to ((Crocodylia,Aves),Testudines) is likely caused by the limits of numerical optimisation in IQ-TREE rather than by topological differentiation. The approximately unbiased SH test shows no evidence in favour of any topology (Table 7).

**Table 7:** Log-likelihoods and confidence levels of three different tree topologies for Aves, Testudines, and Crocodylia

	Log likelihood	Au	Np
((Aves,Testudines), Crocodylia)	−1,266,499.097	0.569	0.511
((Aves, Crocodylia),Testudines)	−1,266,499.559	0.511	0.471
((Crocodylia,Testudines), Aves)	−1,266,511.883	0.043	0.017

The abbreviations Au and Np stand for the approximately unbiased SH test and the bootstrap probability of the selection

## Conclusions

We introduced three new mt models estimated from large mt protein datasets of metazoan, vertebrate, and invertebrate species. Experimental results showed the advantage of the mt new models in inferring phylogenies for both training and testing data in comparison to the existing mt models. The significant likelihood improvement for almost all testing alignments suggests that the new mt models would help find better phylogenies. The phylogenies with the existing mt models may consist of a considerable number of incorrect bipartitions due to their large distances from the best phylogenies.

The low pairwise correlations among mt models for both amino acid frequency vectors and exchangeability rate matrices suggest remarkable varieties of evolutionary processes of different metazoan lineages. This is particularly true for vertebrates and invertebrates, where their models are the most diverse pair. The new mt models are highly specified to the category of the training data and significantly different from the general models. Note that we also applied the approach to estimate mtPro and mtDeu models for Protostomia and Deuterostomia clades, respectively.

Experimental results confirmed the essential role of model selections in inferring phylogenies from mt protein data. As a general rule, the best-fit model for a certain alignment is the new model estimated from the training data of the same category as the alignment. However, we recommend testing all three new mt models for the study of datasets containing diverse metazoan groups, as mtVer and mtInv might fit better than mtMet for the diverse metazoan alignments.

An alternative approach for model selection is to use model averaging method that allows the estimation of phylogenies and model parameters using all available mt models [32]. In addition, the new empirical mt models can be used as prior probability distribution of amino acid substitution rates in Bayesian analyses [33]. As the new empirical models do not explicitly encode site-specific biological constrains, it is worth testing site-heterogeneous models (e.g., LG4X or C60). Finally, mitochondrially encoded proteins are transmembrane proteins with non stationary evolutions, researchers should consider to test transmembrane-specific amino acid substitution models (e.g. PHAT [26]) and non stationary models (e.g. Coala [34]).

The phylogeny of 993 amniote species inferred from mt proteins with the new models shows strong support for the hypothesis that Testudines, Crocodylia, and Aves form one separated clade within amniotes. However, we could not determine precise relationships among Testudines, Crocodylia, and Aves.

## Methods

### Model

We assume the amino acid substitution process to be a general time-reversible process and that the substitution processes of amino acid sites are independent [16]. The amino acid substitution model is characterised by a Markovian substitution matrix, *Q* = {*q*_*x* , *y*_}, that is unchanged during the evolution across all sites. The distribution of amino acid frequencies, ***π*** = {*π*_*x*_}, is also assumed to be stationary (or in equilibrium) and fixed across sites and evolution histories. Moreover, *Q* and ***π*** are dependent, where *Q* ***π*** = 0. Since the process is time-reversible, *Q* = {*q*_*x* , *y*_} can be rewritten as:

$$ {q}_{x, y}={\pi}_y{r}_{x, y}\;\mathrm{and}\;{q}_{x, x}=-{\Sigma}_{x\ne y}{q}_{x, y}, $$

where *r*_*x* , *y*_ = *r*_*y* , *x*_ is the exchangeability coefficient between amino acids *x* and *y*.

Since time and branch lengths are normally measured by the number of mutations, matrix *Q* is normalised such that a time unit is equivalent to one amino acid mutation as follows:

$$ \dot{Q}=\frac{Q}{\mu}\;\mathrm{where}\;\mu =-{\Sigma}_x{q}_{x, x}. $$

The normalisation of *Q* would not affect likelihood values or tree topologies but branch lengths only.

Given normalised matrix *Q*, the probability of amino acid substitutions over the course of time *t* is calculated as:

$$ P(t)={e}^{Qt}, $$

where the right term, *e* ^*Qt*^, denotes the matrix exponential.

The likelihood of phylogeny *T* and matrix *Q* of a given alignment *D* is calculated as:

$$ LK\left( T, Q; D\right)=\prod_i LK\left( T, Q;{D}_i\right), $$

where *D*_*i*_ is the data at site *i* of alignment *D*. In addition, *LK*(*T*, *Q*; *D*_*i*_) can be calculated using the pruning algorithm [35].

It is well known that evolution rates among sites are variant and are best described by a gamma distribution with parameter *α* [36]. The proportion of invariant sites also contributes to the likelihood of a phylogeny. The likelihood of phylogeny *T*, matrix *Q*, rate variants *α*,and the proportion of invariant sites, *ν*, with given alignment *D* can be calculated as follows:

$$ LK\left( T, Q,\alpha, v; D\right)= v{\prod}_i LK\left(\mathrm{Invariant};{D}_i\right)+\left(1- v\right){\prod}_i\frac{1}{C}{\Sigma}_c LK\left({\rho}_c T, Q;{D}_i\right) $$

where *ρ*_*c*_ is the rate of category *c* of the gamma distribution with parameter *α* , and *ρ*_*c*_ *T* is tree *T* with branch lengths multiplied by the factor *ρ*_*c*_.

Many software applications have been developed to estimate *T* , *Q* , *α*, and *v* for a given alignment *D* [22, 37, 38].

Given a set of alignments, **D** = {*D* ^*i*^}, matrix *Q* can be estimated from **D** by maximising the likelihood function as follows:

1 $$ LK\left( Q;\boldsymbol{D}\right)={\prod}_i LK\left({T}^i, Q,{\alpha}^i,{v}^i;{D}^i\right). $$

Le and Gascuel [16] proposed a method to estimate matrix *Q*. First, *T* ^*i*^ , *α* ^*i*^, and *v* ^*i*^ are estimated using an initial matrix *Q*, and subsequently matrix *Q* is estimated based on the newly estimated parameters *T* ^*i*^ , *α* ^*i*^, and *v* ^*i*^. The optimising process is repeated until the likelihood improvement is insignificant.

## Abbreviations

Mt.: Mitochondrial
NCBI: National Center for Biotechnology Information
RF: Robinson-Foulds
